# A One-Step, Streamlined Children’s Vision Screening Solution Based on Smartphone Imaging for Resource-Limited Areas: Design and Preliminary Field Evaluation

**DOI:** 10.2196/18226

**Published:** 2020-07-13

**Authors:** Shuoxin Ma, Yongqing Guan, Yazhen Yuan, Yuan Tai, Tan Wang

**Affiliations:** 1 College of Software Engineering Southeast University Nanjing China; 2 TerryDr Infomation Technology Nanjing China; 3 The Fourth Hospital of Hebei Medical University ShiJiaZhuang China

**Keywords:** vision screening, resource-limited application, photorefraction, strabismus, myopia, anisometropia, mHealth, screening

## Abstract

**Background:**

Young children’s vision screening, as part of a preventative health care service, produces great value for developing regions. Besides yielding a high return on investment from forestalling surgeries using a low-cost intervention at a young age, it improves school performance and thus boosts future labor force quality. Leveraging low-skilled health care workers with smartphones and automated diagnosis to offer such programs can be a scalable model in resource-limited areas.

**Objective:**

This study aimed to develop and evaluate an effective, efficient, and comprehensive vision screening solution for school children in resource-limited areas. First, such an exam would need to cover the major risk factors of amblyopia and myopia, 2 major sources of vision impairment effectively preventable at a young age. Second, the solution must be integrated with digital patient record-keeping for long-term monitoring and popular statistical analysis. Last, it should utilize low-skilled technicians and only low-cost tools that are available in a typical school in developing regions, without compromising quality or efficiency.

**Methods:**

A workflow for the screening program was designed and a smartphone app was developed to implement it. In the standardized screening procedure, a young child went through the smartphone-based photoscreening in a dark room. The child held a smartphone in front of their forehead, displaying pre-entered personal information as a quick response code that duplexed as a reference of scale. In one 10-second procedure, the child’s personal information and interpupillary distance, relative visual axis alignment, and refractive error ranges were measured and analyzed automatically using image processing and artificial intelligence algorithms. The child’s risk for strabismus, myopia, and anisometropia was then derived and consultation given.

**Results:**

A preliminary evaluation of the solution was conducted alongside yearly physical exams in Luoyang, Henan, People’s Republic of China. It covered 20 students with suspected strabismus and 80 randomly selected students, aged evenly between 8 and 10. Each examinee took about 1 minute, and a streamlined workflow allowed 3 exams to run in parallel. The 1-shot and 2-shot measurement success rates were 87% and 100%, respectively. The sensitivity and specificity of strabismus detection were 0.80 and 0.98, respectively. The sensitivity and specificity of myopia detection were 0.83 and 1.00, respectively. The sensitivity and specificity of anisometropia detection were 0.80 and 1.00, respectively.

**Conclusions:**

The proposed vision screening program is effective, efficient, and scalable. Compared with previously published studies on utilizing a smartphone for an automated Hirschberg test and photorefraction screening, this comprehensive solution is optimized for practicality and robustness, and is thus better ready-to-deploy. Our evaluation validated the achievement of the program’s design specifications.

## Introduction

High myopia and amblyopia are 2 major causes of vision impairment, with roots and traits in childhood [1,2]. Compared to other vision disorders, such as glaucoma and macular degradation, the primary risk factors for these 2 disorders are more diagnosable [3]: refractive error can be tracked for both myopia and amblyopia [4]. Additionally for amblyopia, the presence of strabismus can be watched for [5]. Furthermore, the responsiveness of these disorders to simple and cost-effective interventions at early stages [6-8] makes screening the young more valuable. Yet, since the early-stage impacts are subjective, children may not discuss them with their parents and miss the chance to receive cost-effective treatment. This is especially worrisome in developing areas, where the parental generation has a low rate of myopia, and thus poor understanding of its risks, and a high fertility rate thinly divides parental care [9,10]. In fact, the rate of myopia is quickly rising globally, and is rampant not only in eastern Asia but also across the developed world and among all ethnic groups [11]. At earlier stages, mild myopia disrupts daily life and impairs children’s learning capabilities. At later stages, severe myopia has a high possibility of resulting in retinal detachment, a blinding and recurring complication [12], among others [13]. Amblyopia generally does not lead to full blindness but does not respond as well to treatment in adulthood.

In 2019, the Chinese central government announced guidelines to cover 185 million kindergarten to grade 12 students with yearly vision screening, focusing on myopia. The recommended procedure includes an eye chart and automatic refractor exams. In practice, however, a number of problems limited the implementation scale. First, the shortage and uneven distribution of systematically trained optometrists, the qualified examiners, will hardly improve in a few years [14]. Second, physical access to remote areas is difficult and time-consuming, especially with high-precision optical measurement equipment that may not endure the transportation. Last, with benefits taking a decade or longer to materialize, investments of US $10 to $20 per student per year by local governments on the program are prohibitive. As a result, while pilot programs are conducted in richer cities, other areas, especially remote ones, are not following.

Luoyang city in Henan, one of the most populated but less developed provinces, leads experimenting with efficient and scalable vision screening programs. While internet-enabled systems and semiautomated workflows have achieved the expected results in the city centers and suburban towns, costs to cover rural areas are well above budget due to transporting technicians and equipment to reach sparse populations. Although some lightweight alternatives have been proposed and evaluated in academia, they suffer from one or more shortcomings: dependency on a specific device, lack of robustness, and lack of practical workflow integration [15-17]. For example, GoCheck Kids [18-20], one recognized commercial product, requires specific iPhone models.

In this study, a new photoscreening solution for resource-limited areas was developed and evaluated. With a smartphone-based automated Hirschberg test and photorefraction, risk levels of strabismus, myopia, and anisometropia can be detected on the spot. Powered by deep learning and image-processing algorithms, the measurement and analysis procedures are fully automated and robust. By replacing dedicated devices with widely available smartphones and conducting an exam that resembles normal photo-taking, no equipment or trained technician needs to be transported. Following a streamlined workflow, about 200 students can be screened and logged with a compact team of 1 supervising teacher and 1 consulting optometrist equipped with 4 mainstream smartphones and equipment available in a rural school. Evaluation experiments show a sound screening accuracy among primary school students.

## Methods

### Principles of an Automated Hirschberg Test and Smartphone Photorefractory

Strabismus can be roughly defined as misalignment between the binocular axes, or 2 eyes appearing to look in different directions [1]. In a random photo, such gaze directions may rarely be accurately estimated; thus, a photo would not be reliable for determining whether the subject is at risk for strabismus. However, a simple point light source and the corneal luminous reflection it causes can make the problem noticeable.

**Figure 1:**
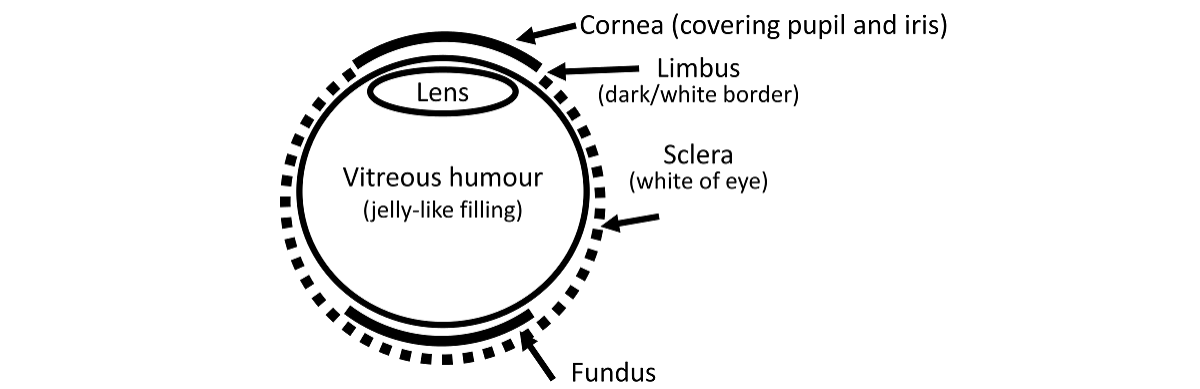
Structure of the eye.

**Figure 2:**
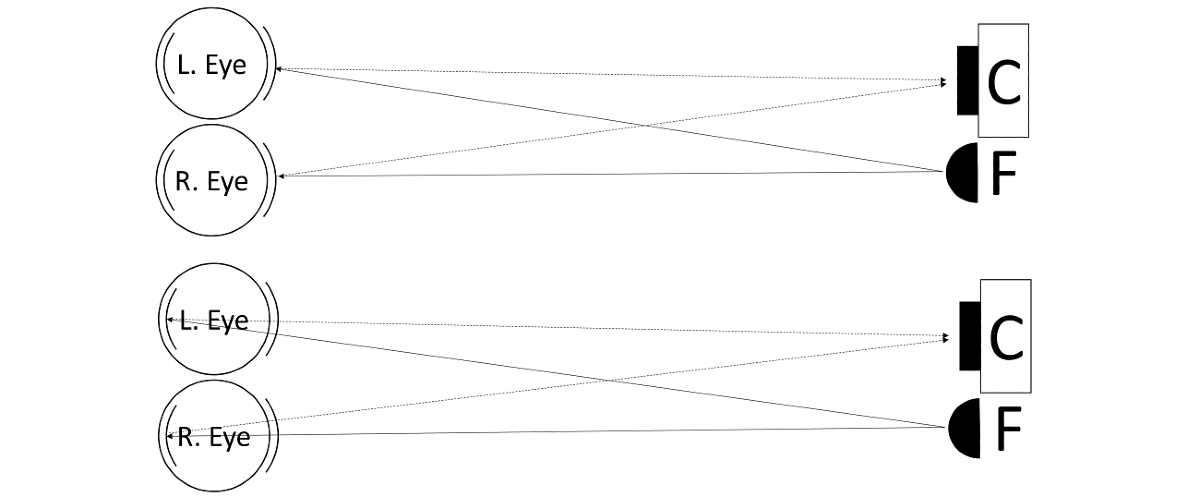
Simplified optical models. Top: Corneal luminous reflection. Bottom: Red reflex. C: camera; F: flash; L: left; R: right.

Although the term “corneal luminous reflection” is technically inaccurate and the optical model is complex [21,22], the from-a-distance image of the corneal luminous reflection of a point light source can be approximated by a bright spot reflected off the surface of the cornea. Simultaneously, the gaze direction of that eye relative to the light source can be roughly reflected by the relative position of the center of the limbus [15], which is attached to the cornea, as illustrated in Figure 1. Combining both models, a simplified automated Hirschberg test can be achieved by taking a photo of the subject’s face with a point flashlight at a sufficient distance and comparing the relative positions of the center of each iris and the bright spot on it [22], as illustrated in Figure 2.

Smartphone photorefractory shares considerable similarity, in principle, with the automated Hirschberg test, but with a twist. They both work by analyzing the reflection pattern of a light source illuminating the eye. However, in smartphone photorefractory, the reflection occurs on the fundus instead of the cornea, and the pattern is primarily linked to the refractive error instead of the ocular axis, as illustrated in Figure 2. With no refractive error, the image of the light source on the fundus would be on its optically conjugate plane, and thus, it would be reflected back following exactly the same path and is almost invisible to the camera. Defocused by refractive error, the illumination would spread over the fundus and be reflected across directions and thus be partially captured by the camera. This is generally known as the red reflex [23].

Additionally, the size of the pupil affects the 2 phenomena. Although it does not interact with corneal luminous reflection, a dilated pupil admits more light into the vitreous chamber [24] and produces a wider viewing angle of the fundus [25]. As a result, when the pupil contracts to a minimal size, fundus reflection is subdued, for a high-quality automated Hirschberg test. When the pupil dilates widely, fundus reflection is prominent and can be better isolated from interfering with corneal luminous reflection. This is a critical consideration in designing the workflow that combines both tests.

**Figure 3:**
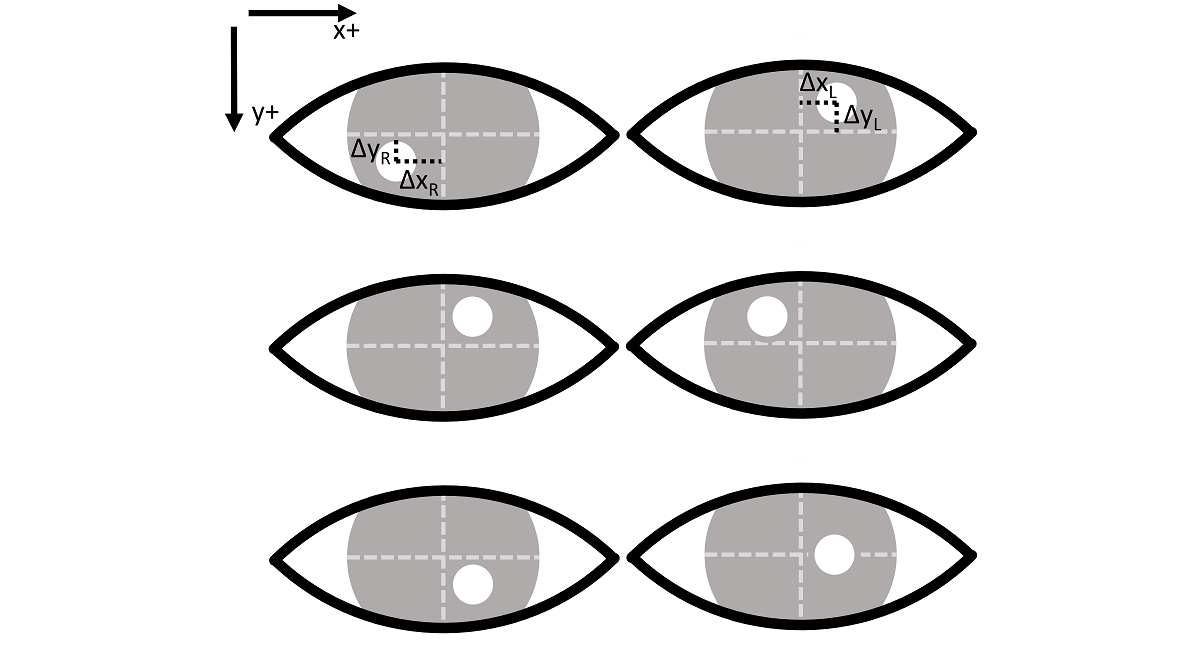
Examples of corneal luminous reflection. Top: Exotropia and vertical strabismus. Middle: Esotropia. Bottom: Vertical strabismus.

The automated Hirschberg test can be numerically processed as follows. After extracting the positions of the limbus and the corneal luminous reflection spot from the captured image, the horizontal and vertical offsets of the center of the corneal luminous reflection spot relative to the center of the corresponding limbus are measured as Δx_L_, Δy_L_ for the left eye and Δx_R_, Δy_R_ for the right eye, respectively. These offsets are measured in pixels and signs following the computer vision convention, as illustrated in Figure 3 (top image). Assuming the frontal plane pixel scale is *α* mm per pixel, the eyeball diameter is *d* mm, and the gap between the camera and the flash is ignorable, the axial deviations of each eye from the camera/flash-ocular axis is as follows:

Θ_horizontal, left_=tan^–1^(2αΔx_L_/d)

Θ_horizontal, right_=tan^–1^(2αΔx_R_/d)

Θ_vertical, left_=tan^–1^(2αΔy_L_/d)

Θ_vertical, right_=tan^–1^(2αΔy_R_/d)

Where tan^–1^() is the inverse tangent function. In this solution, *d* is unmeasurable and is thus set to an average of 20 mm [26]. In summary, the horizontal and vertical binocular axis deviations, measured in angles, are as follows:

Θ_horizontal_=Θ_horizontal, left_–Θ_horizontal, right_

=tan^–1^(2αΔx_L_/d)–tan^–1^(2αΔx_R_/d)

Θ_vertical_=Θ_vertical, left_–Θ_vertical, right_

=tan^–1^(2αΔy_L_/d)–tan^–1^(2αΔy_R_/d)

Intuitively, significantly positive and negative θ_horizontal_ indicate exotropia, as illustrated in Figure 3 (top image) and esotropia (middle image), respectively. A significantly nonzero θ_horizontal_ indicates vertical strabismus, as illustrated in Figure 3 (top and bottom images).

**Figure 4:**
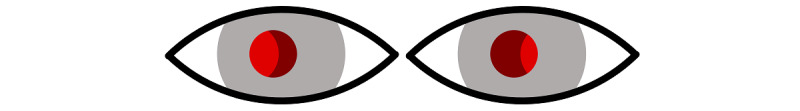
Typical red reflex pattern, with flash on the left. Left: Myopia. Right: Hyperopia.

The red reflex phenomenon, based on which photorefractory is implemented, is hard to robustly quantify with a point light source and uncalibrated parameters [27]. From the examiner’s perspective, assuming the light source is positioned to the left of the camera, a red crescent rising from left side of the pupil is associated with higher degrees of myopia and one from the right side indicates higher degrees of hyperopia, as illustrated in Figure 4. The presence of the crescent can be reasonably reliably detected as an objective and qualifiable signal for screening. The crescent width-severity relationship, however, requires smartphone model–specific calibration and may not hold across examinees; thus, it should be used only as a reference. Additionally, due to the eccentricity of the light source dot, no red reflex can be caused by either refractive error below threshold or a contracted pupil [25,27] (which is hard to detect without close examination). Further, both severe myopia and hyperopia lead to a fully lit-up pupil and are, thus, indistinguishable.

### Core Artificial Intelligence and Image-Processing Algorithms

Although the smartphone-based automated Hirschberg test and photorefraction solution share the same corneal luminous reflection and red reflex principles with specialized devices, it lacks important features, including nonvisible and structured light sources, distance sensors, and a calibrated imaging system. Despite the exam accuracy being inevitably compromised, the applicability of the solution to screening and measurement automation is still obtainable using software. Sequentially in the acquired images, the head tilting and eye locations are detected by facial landmark recognition, the centers of the limbus are estimated by contour detection, the patterns of corneal luminous reflection and red reflex are estimated through shape fitting, and the frontal plane scale of pixels is estimated using an image analysis of the quick response code. It should be noted that processing would be done first on the automated Hirschberg test images and then on the photorefractory images for the former’s higher signal-to-noise ratio, which will be explained in the Discussion section.

#### Head Tilting and Eye Location Recognition

The proposed solution integrates the OpenFace [28] library for facial landmark detection. This deep learning–based library not only produces key point coordinates of each part of the face, it also estimates head gestures and gaze directions, which may support automatic examinee attention analysis and other screening quality controls in later versions. These key points, however, are not positioned to pixel-level accuracy and thus require further processing.

With the key points on the oval contour from OpenFace, closed eyes with a height-to-width ratio below the threshold of η=0.25 can be rejected. In images where both eyes are open, local patches of the image are extracted for hard-coded image processing in later stages. The head tilting angle is recorded to correct coordinates in later processing.

#### Limbus Detection

Ideally, the limbus is a ring that is white on the outside and dark on the inside. In a photo, however, the transition may not be clear-cut, and part of it may be covered. The limbus detection algorithm in this solution addresses both issues with the following steps on each eye.

First, the sclera-limbus-iris region of interest is extracted from the background of nearby skin that matches the hue and saturation of other skin areas. Second, the IsoData automatic thresholding [29] is applied to a histogram of the region of interest to estimate the boundary between the bright sclera and the dark iris. Third, a classic Hough transform of curves [30] between ±45° from a horizontal line is performed to refine the estimated left and right boundary curves without considering the covered parts. Last, using the estimated color contrast between the sclera, iris, and the estimated limbus size from the automated Hirschberg test image, the limbus in the corresponding photorefraction image can be detected with the Hough transform of the same radius. Instead of covering the estimated boundaries, the transform is optimized to maximize contour contrast.

#### Corneal Luminous Reflection and Red Reflex Pattern Analysis

Within the detected limbus, the raw corneal luminous reflection pixels are detected as the cluster of pixels of maximal intensity across all channels, while the raw crescent pixels are filtered out as those with (a) the red channel brighter than green and blue combined, and (b) brightness more than twice the median of that of all pixels the limbus or red channel saturated. An inscribed circle is fitted among the raw corneal luminous reflection pixels, whose center is used to represent the point of corneal luminous reflection. A circumscribed oval is fitted among the raw red-flex pixels, whose width is used to estimate the severity of refractive error. In this experiment, a width over 1 mm is regarded as an indicator of refractive error. These choices of pattern fitting are slightly different from those in previous studies because preliminary research shows that they are more robust on the lower image quality of amateur users.

#### Frontal Plane Pixel Resolution Scale Estimation

In this solution, accurate interpupillary distance is measured for follow-up eyeglass frame selection. A quick response code displayed on a smartphone held close to the examinee’s forehead with its physical size encoded is used as a reference scale. Since the application programming interface of the physical resolution of a screen is available on most smartphones, it offers the same functionality as dedicated tools such as a special glass frame with marks [17] but is universally available. Compared to other everyday-object tools, such as credit cards or rulers, the structured image pattern on a self-illuminating screen guarantees high contrast, which greatly improves accuracy and robustness.

### Solution Design Guidelines and System Outline

As an alternative solution to the existing high-quality, full-service program with an automatic refractor exam and procedures by qualified optometrists, the proposed setup has been adapted to resource-limited rural schools with multiple modifications.

First, the fundamental technology may cover qualitative instead of quantitative analysis on the risk of strabismus, myopia, and anisometropia. Even though earlier research verified that such technology could produce reliable quantitative results, it is limited strictly to specific smartphone models, which are rarely available to the average user. Since the goal is to screen instead of diagnose, ease of use and accessibility dominate over a level of performance higher than necessary.

Second, the workflow should serve children as young as 4 years old. Although the application of smartphone photoscreening has been proven feasible to young children [18], their inability to understand instructions and reluctance to cooperate reduces the chances of successful measurement and thus prolongs measurement time. Therefore, pediatricians are consulted to optimize the workflow for young children’s psychological characteristics.

Third, the procedure should be executable by school teachers under loose supervision of a general practitioner (GP) with only remote training, yet the exam time per child should be no more than 1 minute. Although expertise may not be required to carry out the procedures, legal concerns and local customs require a GP, who services several villages that may be hours of travel apart, to perform the job. Additionally, on-site consultation provided by the GP must be part of the procedure.

Last, data analysis should be mostly automatic and nearly instantaneous. In China, optometric teleconsultation is under-staffed, and few optometrists are qualified to interpret photoscreening images. Giving on-site face-to-face feedback on the screening results not only builds trust and reduces the burden of follow-up phone calls to farmer parents with only a few hours of free time in the evening, it also creates an opportunity for offering other services, such as distributing premade eyeglasses.

**Figure 5:**
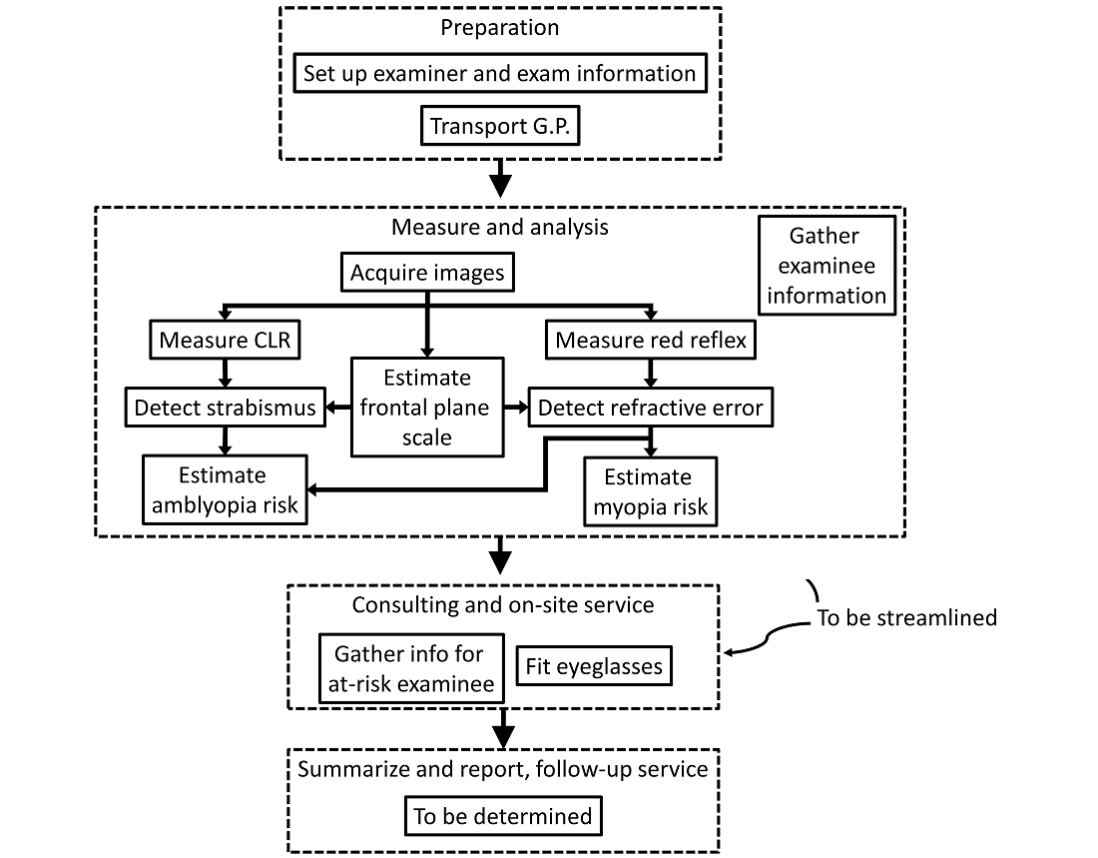
System outline. CLR: corneal luminous reflection; G.P.: general practitioner.

Following the guidelines and the smartphone-based automated Hirschberg test and photorefraction principles, the system is outlined in Figure 5. The core image-processing algorithms are discussed in the previous subsection. The streamlined workflow is explained in the next subsection.

### Streamlined Workflow With One-Step Measurement

**Figure 6:**
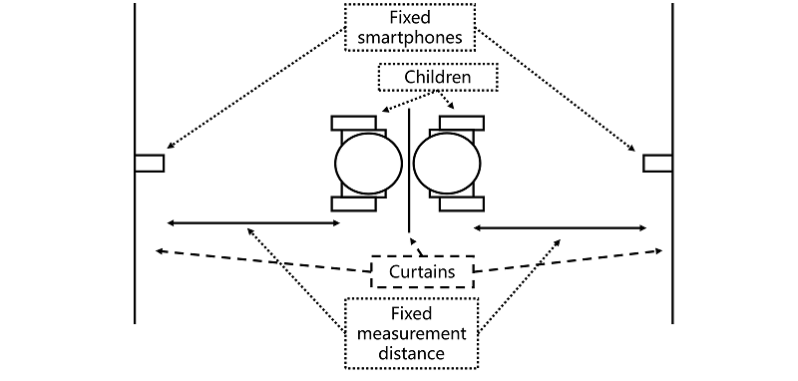
Screening room setup.

Figure 6 shows a typical screening room setup. Two children sit back-to-back with a teacher standing next to them (not illustrated). Dark curtains are hung between the children and behind the smartphones, the former to minimize interference between the 2 parallel tests and the latter to act as projector screens, described next.

**Figure 7:**
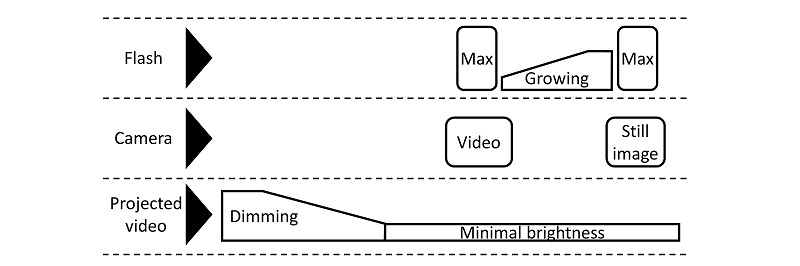
The exam timeline, from an illumination and image acquisition perspective. Max: maximum intensity.

Figure 7 illustrates the exam timeline from an illumination and image acquisition perspective, specifically the coordination of the camera with flash on the smartphone and a video projected onto the curtain in front of the children. When called, the children walk into the room and get seated, then start watching the video projected onto the curtain in front throughout the whole exam, with varying brightness. After several seconds of normal intensity, the video is dimmed and smoothly adapts the children’s eyes to the dark surroundings, thus dilating their pupils for more accurate measurement. After the video reaches its lowest brightness for 5 seconds to induce significant pupil dilation [31], the imaging procedure starts. With the smartphone camera, a short video is recorded during which a flash with maximum intensity is triggered. Followed by 5 seconds of the flash gradually brightening up from minimal in torch mode to contract the pupils in a controlled fashion, a still image is captured with another burst of a maximally intense flash, and the raw data acquisition for 1 child is completed. Since all equipment remains in place and the child sits still through the combined automated Hirschberg test and photorefraction with no intervention required, this is regarded as a one-step measurement. The first frame with maximal exposure of the video and the still image are used for red reflex and corneal luminous reflection pattern analysis, respectively, and the reasoning for this is explained in the Discussion section.

**Figure 8:**
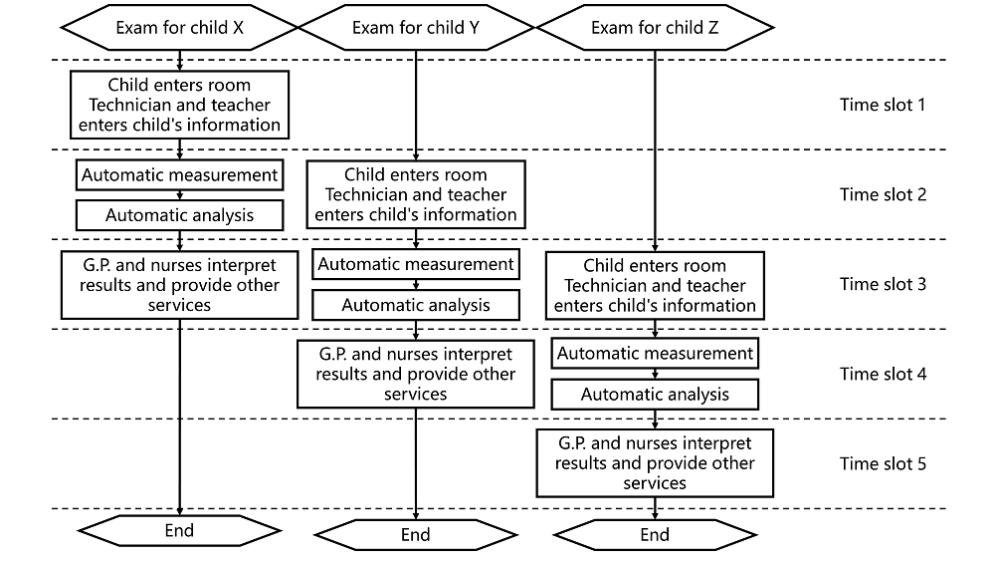
Streamlined workflow for 3 children. G.P.: general practitioner.

Figure 8 illustrates the workflow for serving 3 children in a streamlined fashion. On being called, a child enters the dark room and gets seated while a teacher enters the child’s personal information on a smartphone. After the adaptation period, the child is given the smartphone and instructed to hold it directly in front of their forehead while the image acquisition process begins on the capturing smartphone, as described above. On receiving the automatically analyzed result, a GP or nurse interprets the results to the teacher.

### Clinical Evaluation Setup

To evaluate the solution in real-world environments, experiments were conducted alongside physical exam programs in Luoyang, Henan, between September 1 and October 15. A total of 100 students aged evenly between 8 and 10 years from 2 schools were selected, 20 of whom were suspected by their teacher to have strabismus and the rest were randomly chosen among their classmates. The experiments were conducted in the school infirmaries under direct supervision of either the optometrists from the screening team or the school nurses. The smartphones used for capturing and analyzing data included 1 iPhone 5s (Apple Inc), 1 Honor 8 (Huawei Group Holdings Ltd), and 1 Mi 6 (XiaoMi Corp), all of which were low-end or outdated models at the time.

## Results

### Data Acquisition Efficiency

All 100 screened students successfully completed the data acquisition procedure within 2 rounds, and 87 of them passed with a single attempt. The failures were attributed to random eye blinking. Regardless of individual procedure success or failure, the streamlined workflow in Figure 8 was kept at an even pace, and each time slot took 19 seconds, on average. This translates into almost 200 students screened in 1 hour with just 1 supervising teacher and a consulting optometrist or nurse. With the projected cartoon playing, all students showed a high degree of compliance, and few complained about the exam duration or flashes. Outside the scheduled experiments, some 6-year-old students volunteered to participate, and none exhibited confusion or loss of concentration during the procedure.

### Screening Experiment Results

After the smartphone-based exam, the screened students went through gold-standard optometry exams, and these results were recorded as the ground truth. The gold standard positive thresholds for strabismus, myopia, and anisometropia were chosen as at least 15Δ, at least –0.5D, and at least with a difference of 2D, respectively. The screening positives were defined as a crescent at least 1 mm wide, relative visual angle difference of 10°, and crescent width difference of at least 1 mm, respectively. Using the raw counts of true positives (TP), false positives (FP), true negatives (TN), and false negatives (FN), the statistical metrics accuracy, sensitivity, and specificity were defined as follows:

Accuracy=(TP+TN)/(TP+TN+FP+FN)

Sensitivity=TP/(TP+FN)

Specificity=TN/(TN+FP)

**Figure 9:**
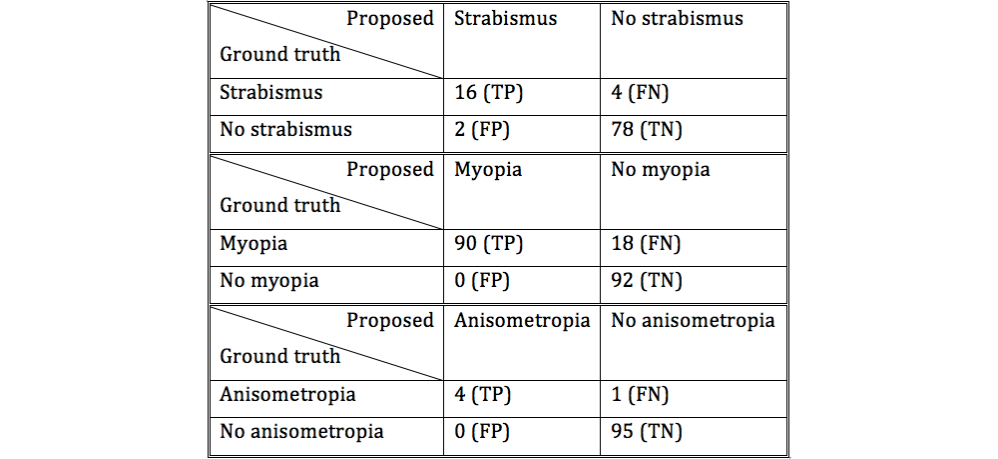
Experiment results: counts. FN: false negatives; FP: false positives; TN: true negatives; TP: true positives.

The raw counts of the experiment are summarized in Figure 9. Since myopia is diagnosed on a per-eye basis, its total count is double the number of examinees. It should also be noted that all 4 false negatives in the strabismus tests were attributed to the weakness of the Hirschberg tests in detecting hidden strabismus. The statistical metrics are tabulated in Table 1.

**Table 1:** Experiment results: accuracy, sensitivity, and specificity.

Metric	Risk factor		
	Strabismus	Myopia	Anisometropia
Accuracy	0.94	0.91	0.99
Sensitivity	0.80	0.83	0.80
Specificity	0.98	1.00	1.00

## Discussion

### Current Limitations

Although the solution produced satisfying accuracy with high efficiency in screening children in resource-limited areas, major improvements should be investigated in follow-up studies. First, the smartphone flash mechanism is unnecessarily counterproductive. The light-emitting diodes for flash used in modern smartphones are specifically designed to work in 2 modes: one consuming very high power in a short burst or limited power continuously. With a fixed exposure time to control motion blur, using the former mode reduces the camera’s sensitivity to light or analog gain and thus improves the signal-to-noise ratio in acquiring both automated Hirschberg test and red reflex images. However, modern smartphones use preflash to induce pupil contraction to suppress the red reflex phenomenon, more generally known as the red-eye effect. To circumvent this, the implementation app replaced the still-image application programming interface with the first intensely illuminated frame extracted from the video. This frame, though, is usually not illuminated with the highest intensity flash, and thus it suffers a reduced signal-to-noise ratio. If a future version of the smartphone camera control provides more flexibility in such control, image quality may be improved.

Second, the implementation software uses a nongeneralized limbus detection algorithm. The proposed method worked robustly on the Chinese students partly because they have a black limbus that is in high contrast to a white sclera. However, on some of the older students who wore colored contact lenses, the low signal-to-noise ratio of the images occasionally caused detection error.

### Potential Future Work

In addition to the factors known to limit the performance of the proposed solution, further exploration is planned to address other areas for improvement. First, on the coordination between the smartphone and the projector: In the current version, these run independently and rely on an operator to synchronize them. A future version may include automatic synchronization on the smartphone side by detecting a predefined pattern of the projector, such as information encoded in color variation.

A second issue of interest is the video played during the exam. Some clips seemed to contain too much activity for some of the younger students to be able to maintain a fixed gaze on a consistent part of the screen, introducing unnecessary change in the visual axes. Some background music seemed better at attracting the students’ attention quickly. Detailed comparison and analysis, however, were beyond the scope of this preliminary study and will thus be left for follow-up studies.

The last area for future work is on carrying out the experiment on a larger scale and with different setups. The experiment reported in this paper was limited to 2 urban schools, and the equipment and personnel execution capability may be different from those in rural schools.

### Conclusion

In this paper, we propose an effective and efficient smartphone-based children’s vision screening solution for areas with limited resources, implemented and evaluated. In a dark room, the examinee watches cartoons projected onto a dark curtain screen with program-controlled intensity, while a smartphone in front of the curtain runs the measurement program. The smartphone’s flash and camera are used as an eccentric point light source and image sensor, respectively, following the automated Hirschberg test and photorefractory principles. In the acquired images, the frontal plane resolution scale is automatically calibrated from a personal information–encoded quick response code displayed on another smartphone held in front of the examinee’s forehead. The examinee’s head gesture and eye location are estimated using a deep learning model. The limbus areas, interpupillary distance, corneal luminous reflection spots, and red reflex areas are detected by their corresponding algorithms to derive risk levels of strabismus, myopia, and anisometropia.

By splitting the workflow into 3 parts of inputting information, acquiring images, and automatically analyzing, interpreting, and consulting, the screening procedure is streamlined such that 1 GP supervising 1 teacher can screen, get an automatic diagnosis, and give on-the-spot consulting to 200 children per hour. The examiners need little training, and the equipment, including the model-neutral mainstream smartphones, is available to the average school. In the evaluation of 100 students between 8 and 10 years old, the high throughput and screening accuracy of the proposed solution were verified and recognized by participating optometrists.

## Abbreviations

FN: false negative
FP: false positive
GP: general practitioner
TN: true negative
TP: true positive
